# Bruton’s tyrosine kinase inhibitors in the treatment of primary central nervous system lymphoma: A mini-review

**DOI:** 10.3389/fonc.2022.1034668

**Published:** 2022-11-17

**Authors:** Jing Shen, Jinghua Liu

**Affiliations:** ^1^ Department of Hematology, Capital Medical University Affiliated Beijing Friendship Hospital, Beijing, China; ^2^ Department of Hematology, The Fifth Medical Center of Chinese PLA General Hospital, Beijing, China; ^3^ Department of Hematology, Northern Theater General Hospital, Shenyang, China

**Keywords:** PCNSL, BTKi, bruton’s tyrosine kinase inhibitors, primary central nervous system lymphoma, ibrutinib

## Abstract

Primary central nervous system lymphoma (PCNSL) is a highly aggressive brain tumor with poor prognosis if no treatment. The activation of the NF-κB (nuclear factor kappa-B) is the oncogenic hallmark of PCNSL, and it was driven by B cell receptor (BCR) and Toll-like receptor (TLR) signaling pathways. The emergence of Bruton’s tyrosine kinase inhibitors (BTKis) has brought the dawn of life to patients with PCNSL. This review summarizes the management of PCNSL with BTKis and potential molecular mechanisms of BTKi in the treatment of PCNSL. And the review will focus on the clinical applications of BTKi in the treatment of PCNSL including the efficacy and adverse events, the clinical trials currently being carried out, the underlying mechanisms of resistance to BTKi and possible solutions to drug resistance.

## Introduction

Primary central nervous system lymphoma (PCNSL) is a rare and highly aggressive non-Hodgkin lymphoma (NHL) with poor prognosis. PCNSL is characterized by specific extra-nodal sites affecting brain and other CNS including spinal cord, eye or cerebrospinal fluid (CSF). It accounts for approximately 4-6% of extra-nodal NHL with a male: female ratio of 1.21:1 (1). PCNSL was recognized as primary diffuse large B-cell lymphoma of the CNS by the World Health Organization (WHO) 2016 classification of hematopoietic and lymphoid tumors (2). The 2022 WHO classification revision arises an entity named as primary large B-cell lymphoma of immune-privileged sites based on their respective anatomical structures including PCNSL, primary vitreoretinal lymphoma (PVRL) and primary testicular lymphoma (PTL) (3). This new entity now combines a group of aggressive B-cell lymphomas which share similar immunophenotypic and molecular features in the immunocompetent older patients with male predominance (4). With the understanding of the pathophysiology of PCNSL, relevant biomarkers from CSF by liquid chromatography/mass spectrometry establish a biological correlation with tumor microenvironment (5). The existence of aberrant somatic hypermutation mutation analyses in CSF showed tumor-associated genes may provide an alternative pathway for PCNSL development (6). The consequences of potential pathogenetic and relevant genomic landscape in PCNSL have been uncovered in recent years (7). Based on this background, we focus on a new potential oral inhibitor of Bruton’s tyrosine kinase (BTK), which has been explored as a novel therapeutic option against CNS lymphomas.

B-cell receptor (BCR)/NF-κB (nuclear factor kappa-light-chain-enhancer of activated B cells) signaling axis supports the survival and proliferation of malignant B cells (8). BTK is centrally involved in BCR/NF-κB signaling pathway and BTK inhibitors (BTKis) were developed as promising novel agents of B-cell lymphoma (9). BTK contains five domains and all five approved BTKis (ibrutinib, acalabrutinib and zanubrutinib, tirabrutinib and orelabrutinib) target cysteine 481 (C481) domain (10). Ibrutinib is a first-generation BTKi, and it was approved for the treatment of chronic lymphocytic leukemia (CLL), mantle cell lymphoma (MCL), marginal zone lymphoma (MZL), chronic GVHD (graft-versus-host disease) and Waldenström’s macroglobulinemia (WM) by the FDA (U.S. Food and Drug Administration) (11). However, in real world studies, estimated 19-49% of patients discontinued ibrutinib, off-target binding was the most common reason for discontinuation (12). To reduce toxicity and side effects, next-generation BTK inhibitors improve the tolerability and reduce bleeding and cardiac arrhythmia with more selective kinase inhibition. Acalabrutinib and zanubrutinib were approved by FDA in 2017 and 2019, respectively and zanubrutinib was granted conditional approval in 2020 in China, while tirabrutinib was approved only in Japan (13). Unfortunately, BTK resistance limited the long-time use in B-cell malignancies, and the most common acquired resistance caused by BTK C481 point mutation and phospholipase C gamma 2 (PLCG2) mutation (14, 15). To overcome resistance, noncovalent and reversible BTKis including vecabrutinib and LOXO-305 or ARQ-531 were found to be effective despite presence of mutations within C481S or PLCG2 (16, 17). Some studies show that primary resistance to BTK inhibitors is due to epigenetic rather than genetic changes (14). BTK inhibitors have been investigated in the treatment of CNS lymphomas in clinical trials and showed promising therapeutic options (18, 19). Based on impressive responses, National Comprehensive Cancer Network (NCCN) guidelines were updated in 2018 to include BTKi for the management of relapsed/refractory (R/R) PCNSL (20). Tirabrutinib is the first approved BTKi for the treatment of R/R PCNSL in Japan (21, 22).

The present review will focus on potential molecular mechanisms and the applications of BTKis in PCNSL. This review includes five main topics: i) an update on management of PCNSL; ii) the BCR/BTK signaling pathway in PCNSL; iii) the clinical application of BTKi in PCNSL; iv) the mechanism of resistance to BTKi and management; v) the side effects of BTKi.

### Overview of PCNSL

Most PCNSLs (over 90%) are histologically classified as DLBCL, followed by rare Burkitt, lymphoblastic T-cell and low-grade lymphomas (23, 24). Imaging choice in brain tumor including magnetic resonance imaging (MRI) or 18F-fluorodeoxyglucose (FDG) positron emission tomography (PET) is crucial for diagnosis and surgical planning (25, 26). Noninvasive imaging detect provides prognostic insight and minimal residual disease (MRD) assessment as well (27, 28). Typical brain parenchyma involvement is found in more than 90% of the cases, and most frequent locations are cerebral hemispheres, corpus callosum and deep gray matter (29, 30). leptomeningeal or ocular involvement is detected in 10-20% of patients at diagnosis (31). A systematic review of 1481 patients with PCNSL showed brain biopsy was the preferred method of diagnosis in 95% of patients, and preoperative CSF analysis obviated 7.4% of cases suffering surgery (32).

According to the Hans algorithm, most PCNSLs are classified as non-germinal center B-cell-like (non-GCB) phenotype (78-96%), which could interpret the poor prognosis in PCNSL (33–35). By using immunohistochemistry (IHC), double-expressing lymphomas (DELs) are defined as co-expression of 2 oncogenes (MYC and BCL2) (36). The prognosis of PCNSL patients with DEL was controversial (37, 38). Although they reported that patients with MYC expression or BCL2 expression were significantly associated with poor overall survival (OS) respectively (37, 38). A meta-analysis of the literature showed a significantly high frequency of concurrent mutations with MYD88 L265P and cluster of differentiation 79B (CD79B) in PCNSL (39). MYD88 L265P mutation is most common mutation associated with activated B-cell-like (ABC) subtype DLBCL arising in immune-privileged sites. In mutational spectrum analysis of PVRL using next-generation sequencing (NGS), MYD88 and CD79B mutations were reported in 74% and 55% of patients, respectively. There is a considerable overlap between PCNSL/PVRL and MCD genetic subtype (based on the co-occurrence of MYD88 and CD79B mutations) DLBCL (40). PD-L1 amplifications in PCNSLs were identified and PD-1/PD-L1 inhibitors probably will be a treatment option (41). In Nayyar N et al. study, PD-L1 expression was detected in 30% patients using whole-exome sequencing (WES), which may be a mechanism for immune evasion in PCNSL (42).

With the elucidation of the molecular properties, there has been significant progress in the management of PCNSL during the last two decades. There is no standard induction or consolidation treatment for PCNSL patients due to the paucity of phase 3 randomized clinical study. The optimal treatment strategy for PCNSL includes induction, consolidation and/or and maintenance therapy (43). Age and performance status (PS) should be considered in the choice of treatment and are prognostic factors as well (44). High-dose methotrexate (HD-MTX)-based chemotherapy is the standard initial induction treatment (45, 46). Polychemotherapy regimens including HD-MTX plus cytarabine/thiotepa/temozolomide provided improved treatment outcomes compared to MTX monotherapy (47, 48). The progression-free (PFS) and OS were significantly better in patients undergoing upfront autologous stem cell transplant (ASCT) as consolidation strategy, which might be especially beneficial for high-risk PCNSL patient (49). Whole brain radiation therapy (WBRT) followed by HD-MTX only achieved the PFS benefit, but is associated with long-term neurotoxicity (50, 51). Unfortunately, relapse rate is high particularly in elderly or frail high-risk PCNSL patient within the first two years after diagnosis (50). Maintenance therapy is also being explored in clinical trials in elderly or relapsed patients (52). Maintenance group with HD-MTX every three months improved OS compared with HD-MTX followed by WBRT (53). Lenalidomide maintenance delays WBRT in relapsed PCNSL (54).

Although improved treatment response has been achieved in the PCNSL patients, but the management of R/R PCNSL remains difficult. The small-molecule targeted agents have been investigated with identification of the molecular properties in many R/R PCNSL. Immunomodulatory imide drugs (IMiDs) have been investigated in PCNSL due to dose-dependent CSF penetration of IMiDs (55, 56). Inhibitors for the phosphatidylinositol-3 kinase (PI3K)/AKT/mTOR pathway had synergistic effects with BTK inhibitor in a preclinical study (57). Immune checkpoint inhibitors (ICIs) could be possible therapeutic options. Pembrolizumab was shown encouraging outcomes achieving CR in three of five patients, and PFS >13 months (58). Anti-CD19 Chimeric antigen receptor T (CAR-T) cells yielded significant potential and treatment responses in R/R PCNSL (59, 60). With the development of targeted therapies, emerging data demonstrate BTK inhibitors are very promising treatment strategy targeted BCR/BTK signaling pathway (61, 62).

### BCR/BTK signaling pathway in PCNSL

PCNSL is characterized by aberrant activation of BCR/NF-κB and Toll-like receptors (TLR)/NF-κB signaling pathways (40). These two signaling axises have been identified as central signaling pathways in PCNSL and potential targets for molecular therapy (63). Besides these, immune escape, PI3K/AKT/mTOR and JAK/STAT signaling pathways are important mechanisms in R/R PCNSL, which could be the targets of BTKi resistance treatment. MYD88 mutation plays a crucial role in TLR signaling pathway activation, which could activate NF-κB via interleukin-1 receptor-activated kinase 4 (IRAK4) (64). BCR signaling pathway is activated via BTK phosphorylation after binding of the antigen to the extracellular domain of CD79B (65, 66). The activation of both pathways could lead to increased NF-κB signaling. Caspase recruitment domain family member 11 (CARD11) is a downstream member of BCR signaling pathway. CARD11 mutation may contribute to NF-κB and associate with the resistance to single-agent ibrutinib (42). BTKi targeting BCR/NF-κB pathway has led to breakthrough treatment in PCNSL. Targeting PI3K may downregulate upstream pathway. Proteosome inhibitors and IMiDs may prevent release of NF-κB function, and affect downstream pathway. Anti-CD79B CAR-T cells and anti-CD79B antibody-drug conjugates (ADCs) have been investigated as the new therapeutic approaches targeting CD79B (67, 68).

BCR/NF-κB and TLR/NF-κB pathways gene mutations could be identified by different sequencing projects in PCNSL, and also have association with clinical characteristics. CD79B and MYD88 mutations are more frequently observed in PCNSL. Two previous studies involving a large number of PCNSL patients reported mutations in CD79B (83%) and MYD88 (79%) in 71 patients, and mutations in CD79B (41%) and MYD88 (58%) in 177 patients, respectively (69, 70). MYD88 mutation has been found in 38-79% of PCNSL patients, and CD79B mutation was reported in 30-83% of PCNSL patients (65, 69–75). Overall, BCR/NF-κB and/or TLR/NF-κB signaling pathways were altered in >90% of PNCSL patients (71). CARD11 mutation was found in about 11-30% of cases of PCNSL (70, 71). Genetic alterations in PCNSL suggested similar pathogenesis in immune-privileged sites including PTL and PVRL (40, 41, 72).

#### BTK inhibitors in the treatment of PCNSL

##### Ibrutinib in the treatment of R/R PCNSL

In 2017, ibrutinib is used for the first time in the treatment of 18 patients with R/R PCNSL (76). The dose of ibrutinib was 560-840 mg, and it was treated with DA-TEDDi-R (rituximab, liposomal doxorubicin, temozolomide, etoposide and dexamethasone). Efficacy above complete response unconfirmed (CRu) was achieved in 12 patients, and eight patients remained in remission until 15.5 months. The median PFS was 15.3 months, and median OS was not reached (76). In the same year, a phase I study of ibrutinib monotherapy (560-840mg) for the treatment of 13 patients with R/R PCNSL (70). Three patients were refractory to prior treatments including HD- MTX-based chemotherapy and radiotherapy. Clinical responses were achieved in 10 patients, with 5 patients in complete response (CR) and 5 patients in partial response (PR). The median follow-up time was 479 days, and the median PFS and OS was 4.6 months and 15 months, respectively (70). One year later, the same research team reported a phase II study of ibrutinib monotherapy (560-840mg) for the treatment of 29 patients with R/R PCNSL (77). The median follow-up time was 22 months, and the overall response rate (ORR) was 81%, the median PFS and OS was 4 months and 19.5 months, respectively (77). Considering clinical responses of ibrutinib monotherapy are often transient or incomplete, the research team conducted three phase I trials of ibrutinib combined with chemotherapy for the treatment R/R PCNSL. One is rituximab and HD-MTX in combination with ibrutinib (560-840mg) in 9 patients, the ORR was 89%, while 50% of patients had no disease progression after 19.7 months of follow-up (78). The second trial is the ibrutinib (560mg) combined with pan-PI3K inhibitor copanlisib in 6 patients. After a median follow-up of 180 days, the ORR was 67%, including 1 CR, 3 PR, 1 SD (stable disease) and 1 PD (progressive disease) as best response (79). The third trial is designed as ibrutinib (560-840mg) in combination with lenalidomide and rituximab for the treatment of 15 patients with R/R PCNSL or secondary CNSL (SCNSL). 11 patients achieved clinical response, including 4 CR, 7 PR, 2 SD and 1 PD, and while 50% of patients were disease free at 3.03 months after 6.9 months of follow-up (80).

In the same time, the lymphoma study association (LYSA) and the French oculo-cerebral lymphoma (LOC) network reported a phase II trial of 44 patients with R/R PCNSL or PVRL treated with ibrutinib (560mg) monotherapy (18). 27 patients obtained disease control after 2 months of treatment, including 10 CR, 17 PR and 5 SD. The median follow-up time was 25.7 months, and the median PFS was 4.8 months, while 50% of patients survived at 25.7 months (18). Subsequently, a phase I trial reported ibrutinib was combined with temozolomide, etoposide, liposomal doxorubicin, dexamethasone and rituximab (TEDDI-R) in 13 patients with R/R PCNSL. 92% patients responded after receiving only 1 cycle in evaluable 12 patients. 8 patients achieved CR after at least 4 cycles and the remaining 4 patients were continuing treatment. After a median following-up of 5.2 months, the 1-year PFS and OS estimated was 60.0% and 100%, respectively (81). And a retrospective study of 5 patients with R/R PCNSL treated with ibrutinib monotherapy or ibrutinib-based chemotherapy (82). Although 4 patients underwent ASCT, the ORR was 80% and 2 patients received ibrutinib maintenance after achieving CR (82). In another two retrospective studies, patients were treated with ibrutinib (560mg) in combination with chemotherapy. Although the ORR was more than 80%, the median PFS was only 6 months (83, 84). Then in a prospectively registered 14 patients with R/R PCNSL, 11/14 patients were refractory to first line treatment, 5/14 patients had previously received ASCT. All 14 patients received the regimen of ibrutinib, rituximab and lenalidomide (IR2), and achieved 4 CR, 4 PR, 3 SD, 3 PD (85). In another prospective data from phase I/II clinical trials, 33 patients with R/R PCNSL (n=9) or SCNSL (n=24) received ibrutinib monotherapy or ibrutinib-based chemotherapy. Four of 9 patients achieved CR and the median PFS and OS were both 3.1 months (86). Based on a single center experience of single-agent ibrutinib for the treatment of R/R PCNSL, ibrutinib could be an effective bridge-to-transplant treatment. Two of 3 patients achieved CR after receiving ibrutinib, and were eligible for ASCT (87). The published studies with ibrutinib monotherapy and combination treatment for R/R PCNSL were summarized in **Table 1**.

**Table:** Table 1 Summary of published and ongoing ibrutinib monotherapy and combination treatments for R/R PCNSL or newly diagnosed PCNSL.

NCT#/publication	Phase	Cases/Target Enrollment number (n)	Enrolled patients	Regimen
(18)	II	44	R/R PCNSL, PVRL	ibrutinib
(70)	I	13	R/R PCNSL	ibrutinib
(76)	Ib	18	R/R PCNSL	ibrutinib, rituximab, liposomal doxorubicin, temozolomide, etoposide and dexamethasone
(77)	II	29	R/R PCNSL	ibrutinib
(78)	I	9	R/R PCNSL	ibrutinib, rituximab and methotrexate
(79)	I	6	R/R PCNSL	ibrutinib and copanlisib
(80)	I	15	R/R PCNSL	ibrutinib, rituximab and lenalidomide
(81)	I	13	R/R PCNSL	ibrutinib, rituximab, liposomal doxorubicin, temozolomide, etoposide and dexamethasone
(85)	I	14	R/R PCNSL	ibrutinib, rituximab and lenalidomide
(86)	I/II	9	R/R PCNSL	ibrutinib-based chemotherapy
(88)	I	16	newly diagnosed PCNSL	ibrutinib, rituximab and methotrexate
(89)	II	33	newly diagnosed PCNSL	ibrutinib, methotrexate and temozolomide
NCT03581942	Ib/II	45	newly diagnosed PCNSL R/R PCNSL	ibrutinib and copanlisib
NCT02315326	I/II	109	newly diagnosed PCNSL R/R PCNSL	ibrutinib, rituximab, methotrexate, vincristine and procarbazine
NCT03703167	Ib	25	R/R PCNSL	ibrutinib, rituximab and lenalidomide
NCT04421560	Ib/II	37	R/R PCNSL	ibrutinib, pembrolizumab and rituximab
NCT04066920	II	30	R/R PCNSL	ibrutinib, ifosfamide, etoposide and rituximab
NCT02203526	I	93	newly diagnosed PCNSL	ibrutinib, temozolomide, etoposide, doxil and dexamethasone
NCT04129710	II	120	R/R PCNSL	ibrutinib, methotrexate, rituximab, etoposide and lenalidomide
NCT02623010	II	30	newly diagnosed PCNSL	ibrutinib maintenance
NCT04514393	II	33	newly diagnosed PCNSL	ibrutinib, methotrexate and temozolomide
NCT03770416	II	40	R/R PCNSL	ibrutinib and nivolumab
NCT04446962	Ib/II	128	newly diagnosed PCNSL	ibrutinib, rituximab, methotrexate, procarbazine and vincristine

##### Ibrutinib in the treatment of newly diagnosed PCNSL

A retrospective analysis reported ibrutinib in combination with MTX for the treatment of 11 patients with newly diagnosed PCNSL. 9 patients achieved response, including 7 CR and 2 PR. After a follow-up of 11.6 months, the median PFS was 7.4 months while the median OS was not reached (90). A phase I study investigated the combination of ibrutinib, rituximab and HD-MTX (I-RM) for the treatment of 16 newly diagnosed PCNSL patients. 15 patients achieved response, including 8 CR, 7 PR and 1 PD. The median PFS and OS were not achieved after a median follow-up of 24.5 months (88). A phase II study explored the ibrutinib in combination with HD-MTX and temozolomide in 33 newly diagnosed PCNSL patients. 9 patients were enrolled until April 2021. At a median follow-up of 7 months, the 1-year PFS and 1-year OS were 88.9% and 100%, respectively (89). Another phase II trial evaluated ibrutinib maintenance therapy in elderly newly diagnosed PCNSL patients after achieving PR or CR. Four patients with PR improved to CR after maintenance therapy. At median follow-up of 29 months, the 2-year PFS and 2-year OS were 72.6% ± 10.6% and 89% ± 7.5%, respectively (91). With the continuous encouraging study reports, there are still ongoing clinical trials for the treatment of PCNSL with ibrutinib (**Table 1**).

##### Zanubrutinib in the treatment of PCNSL

Zanubrutinib is a second-generation BTKi. The first retrospective study evaluated the efficacy of zanubrutinib-containing regimens in 4 patients with newly diagnosed and 4 patients with R/R PCNSL. All newly diagnosed PCNSL patients achieved CR and 75% of R/R PCNSL patients achieved CR (92). Another retrospective study reported 3 newly diagnosed and 8 relapsed patients with PVRL treated with zanubrutinib monotherapy. At a median follow-up of 7.5 months, 4 patients had disease progression and 7 patients achieved durable CR (93).

##### Acalabrutinib in the treatment of PCNSL

Acalabrutinib is another second-generation BTK inhibitor. Although there are no data about acalabrutinib in PCNSL, there are four ongoing studies. One (NCT04688151) is a phase I trial of dose escalation of acalabrutinib combined with durvalumab in R/R PCNSL or SCNSL. The second (NCT04462328) is a phase Ib trial of acalabrutinib, rituximab and durvalumab in R/R PCNSL. The other two ongoing trials (NCT04906902, NCT04548648) involve single-agent acalabrutinib in R/R PCNSL.

##### Orelabrutinib in the treatment of PCNSL

Orelabrutinib is second-generation irreversible BTKi with high CSF/plasma ratio (94). A retrospective analysis evaluated the efficacy and safety of orelabrutinib-based chemotherapy in 15 patients with R/R PCNSL. 86.7% patients achieved treatment response and the CR rate was 73.3% (95). Another retrospective analysis of orelabrutinib-based regimen for the treatment of PCNSL (newly diagnosed or R/R). 4/4 patients with newly diagnosed PCNSL achieved response, and were in remission until 6 months. 60% (9/15) patients with R/R PCNSL achieved response, and the 6-month PFS and OS rate were 67.7% and 70%, respectively (96).

In a phase II trial of orelabrutinib in combination with anti-PD-1 monoclonal antibody in R/R PCNSL patients, 61.5% patients achieved response, 1-year PFS rate estimated was 66.7% (97). In addition, there are three ongoing studies, one (NCT04961515) is a phase Ib/II study of orelabrutinib combined with sintilimab in R/R PCNSL, another (NCT05021770) is a phase Ib/II study of orelabrutinib combined with thiotepa in R/R PCNSL, the third (NCT05209620) is a single arm phase II study to explore the combination of orelabrutinib and pemetrexed for the treatment R/R PCNSL.

##### Tirabrutinib in the treatment of PCNSL

Tirabrutinib is a second-generation BTK inhibitor. In March 2020, tirabrutinib was approved for the treatment R/R PCNSL patients. Takeshi et al. reported a patient had acute progression of PCNSL after tirabrutinib treatment and he was recovered from coma with 3 days methylprednisolone treatment. MRI lesion in the right thalamus disappeared after the treatment of 3 months (98). Then a phase I/II study reported tirabrutinib in 44 patients with R/R PCNSL. 64% patients achieved response and median PFS was 2.9 months, while median OS was not reached (99). Noriharu et al. reported a case of relapsed PCNSL treated with tirabrutinib. The brain lesion was ameliorated after four-week tirabrutinib treatment. The patient achieved CR after ten- week treatment (100). Subsequently, Hiroko et al. reported a PVRL patient refractory to HD-MTX-based chemotherapy and whole-brain radiotherapy. Tirabrutinib was selected as salvage treatment for this patient. Surprisingly, after two weeks, MRI showed total tumor removal and complete disappearance of the peritumoral signal abnormality (101).

#### BTK inhibitors resistance and management

**Figure 1:**
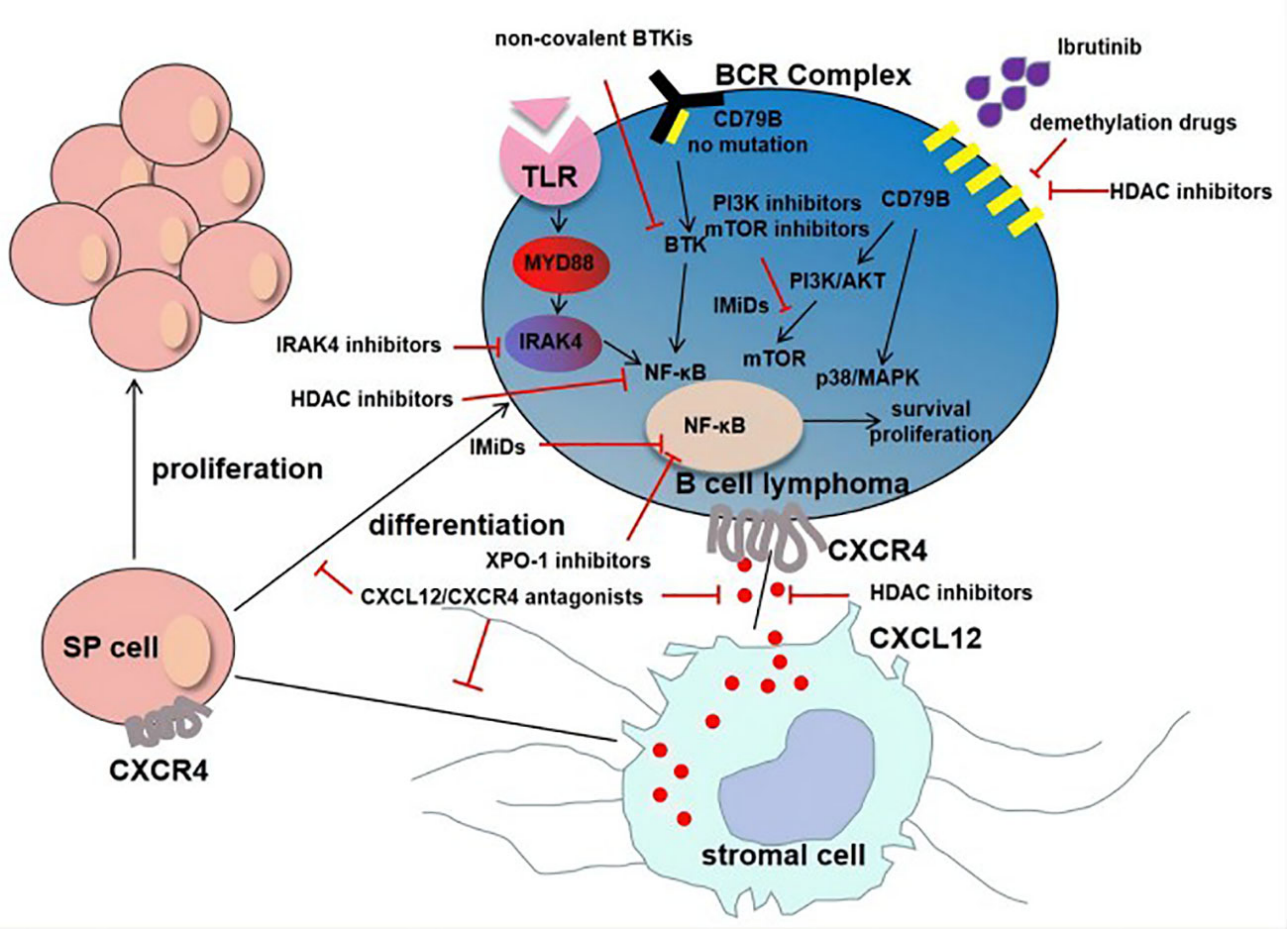
Mechanisms of ibrutinib resistance in PCNSL. For tumor cells without CD79B mutation, ibrutinib resistance was mainly due to MYD88-dependent survival signaling pathway (MYD88→IRAK4→NF-κB). Another important mechanism of resistance is adaptive activation of alternative signaling pathways involving CD79B overexpression and the activation of PI3K/Akt/mTOR pathway and p38/MAPK. The interaction of CXCL12 secreted by stromal cells and CXCR4 secreted by tumor cells promotes tumor growth and resistance. In addition, the existence of SP cells and extrinsic tumor microenvironment are also the mechanisms of drug resistance.

Although the efficacy of BTKi in the treatment of PCNSL is promising, for ibrutinib, about 1/3 of patients develop primary drug resistance, while a large proportion of patients develop acquired drug resistance (102). CARD11 mutation is a known primary ibrutinib resistance mechanism in PCNSL (70). Resistant mutation is reported in CLL and MCL including C481S and R665W mutations in BTK and PLCG2 mutation in the downstream of BTK. Drug resistance is also evolved by amplification of alternative signaling pathways, cancer stem cells (CSCs) and extrinsic tumor microenvironment (TME). Mechanisms of ibrutinib resistance could be as follows (**Figure 1**).

##### Genetic mechanisms and BTKi resistance

Mutations in BTK, PLCG2 and CARD11 have been found in MCL, WM and CLL during ibrutinib therapy, and these are key genetic mechanisms. Tumors without CD79B mutation revealed that MYD88-dependent survival signaling was the main signaling for these tumors in PCNSL (103). A subsequent study revealed that there was a high frequency mutation of KLHL14 in ABC-DLBCL, which promoted assemblage of the MYD88-TLR9-BCR complex, eventually induced resistance to ibrutinib (104).

##### Non-genetic adaptive mechanisms of BTKi resistance

Previous studies have shown that amplification of alternative signaling pathways is closely related to acquired drug resistance. The study of Kim et al. revealed that ibrutinib- resistance ABC-DLBCL cell lines could be generated through co-culture with ibrutinib (105). Moreover, transcriptomic analysis revealed the CD79B overexpression and the activation of PI3K/Akt/mTOR pathway and p38/MAPK in ibrutinib-resistance cell lines (105). BCL2, MYC and XPO1 overexpression may rescue the absence of BCR activity and lead to B cell survival despite BTK inhibition (106).

##### TME and BTKi resistance

TME is a special environment for tumor cells to survive, containing a variety of cells (fibroblasts, endothelial cells, immune cells, etc.) and biochemical factors (cytokines, growth factors, chemokines, etc.) system. Mesenchymal stromal cells (MSCs) are multipotent and major constituent of stromal niches of TME in various tissues and organs. The interaction of tumor cells and the TME mediates the development of drug resistance. Ibrutinib resistance involving TME has been reported in CLL and MCL (107–109). CXCR4 is overexpressed on Tregs and CXCR4 antagonists favor T effector access to TME. Targeting CXCR4 and PD-1 synergistically reduced cell growth in murine tumor models (110).

##### Side population (SP) cells and BTKi resistance

CSCs exhibit stem cell function, including the ability of self-replication and multiple cell differentiation. Previous studies have identified SP cells in various malignancies, which are CSC-like cells and resistant to radiation and chemotherapy. SP cells could be detected in Hodgkin lymphoma, DLBCL and follicular lymphoma (FL) cell lines (111, 112). There was an interaction between SP cells and TME through CXCL12/CXCR4 signaling, which may allow SP cells to evade chemotherapy and targeted therapy (112). CXCL12/CXCR4 antagonist could be an attractive approach to target SP cells (112).

##### Management of BTK inhibitor resistance

Many studies have been performed to explore how to overcome resistance to BTKi in hematological malignancies. Our question arising from this section is whether the new investigational agents penetrate the CNS blood‐brain barrier (BBB). The outcomes and clinical benefits are investigated in patients receiving novel targeted therapeutic approaches and immune checkpoint inhibitors. Optimizing combination of active therapies could be a challenge in the management of BTki resistance.

##### PI3K/AKT/mTOR inhibitors

An acquired ibrutinib resistance could be the activation of alternative pathways independent of BCR. Then Kapoor et al. identified that ibrutinib resistance can be overcome by PI3K isoform inhibitors and the mechanism was targeting the activation of PI3K/AKT/mTOR signaling pathway (113, 114). CARD11, CD79A/B, TNFAIP3, and MYD88 are BCR signaling components, mutation of any of them would drive alternative signaling pathway activation. PI3K isoform inhibitors shrank the tumor by targeting the PI3K pathway in ibrutinib-resistant ABC-DLBCL in vivo models (115). GDC-0084, a small molecule inhibitor of PI3K and mTOR, was detected that its total and free drug of brain tumor tissue/plasma ratio was>1 and>0.5, respectively, which suggested that it can cross BBB (116). Temsirolimus is a mTOR inhibitor which was used in the treatment of 37 patients with R/R PCNSL and achieved response in 20 patients including 5 CR, 3 CRu and 12 PR (117).

##### Reversing the reduced BTK expression

Jain et al. observed the total BTK expression of ABC-DLBCL lines was significantly reduced after chronically exposure to ibrutinib (114). Down-regulation or loss of drug-target expression is another way of acquired drug resistance of tumor cells. The possible mechanisms are the selective reduction of BTK expressing cells and the epigenetic changes due to long-term exposure to ibrutinib (118). Therefore, trying to cooperate with demethylation drugs or deacetylase inhibitors may increase the expression of BTK.

##### BCL2 inhibitors

Jain et al. reported that BCL2 expression could be upregulated in resistant cells under chronic ibrutinib exposure (114). Kuo et al. also have confirmed that synergistic anti-proliferative activity of ibrutinib and BCL2 inhibitor venetoclax in resistant ABC-DLBCL lines (119). It is believed that the venetoclax with the molecular weight of 868.44 may not cross BBB, but Ahmed et al. have confirmed that it can pass through the BBB in 66 samples of relapsed or refractory acute myeloid leukemia or acute lymphoblastic leukemia (120).

##### IRAK4 inhibitors

IRAK4 belongs to the IRAK family and plays an important role in tumor growth and progression. IRAK4 is a TLR adapter protein and activation of TLR/NF-κB signaling pathway is mediated by IRAK4 kinase. The study of Kelly et al. and Schaffer et al. revealed the synergistic anti-proliferative activity of IRAK4 inhibitor and ibrutinib in ABC-DLBCL cell lines with MYD88 mutation (121, 122). There is an ongoing clinical trial of CA-4948 (an oral IRAK4 inhibitor) combined with ibrutinib (NCT03328078) in patients with R/R hematologic malignancies, of which part B included the newly diagnosed PCNSL and ibrutinib-resistance R/R PCNSL (123).

##### Histone deacetylase (HDAC) inhibitors

In ABC-DLBCLs, primary ibrutinib resistance could be induced by MYD88 mutation, Mondello et al. reported that HDAC inhibitor panobinostat could control transcriptionally the expression of MYD88 and synergize with ibrutinib in ABC-DLBCL cell lines with MYD88 mutattion (124). Singleton et al. found that water-soluble panobinostat intraparenchymal administration could be effective in the CNS tumor via surgically implanted microcatheters (125).

##### Bromodomain and extraterminal domain-containing proteins (BETs) inhibitors

JQ1, a selective BET-inhibitor, has shown some synergistic activity with BTKi in ABC-DLBCL (126). BRD4 belongs to BET family member and preclinical studies with BRD4 inhibitors demonstrate the suppression of MYC expression in MYC-driven Burkitt’s lymphoma cell lines (127). JQ1 has been reported quantification of 5 and 0.5 ng/mL for plasma and brain microdialysate due to good CNS penetration in the mouse model (128).

##### Non-covalent (Reversible) BTK inhibitors

Pirtobrutinib is highly potent non-covalent BTKi and inhibits both wild type and C481-mutated BTK due to reversible bind. Some clinical trials demonstrated promising efficacy of pirtobrutinib in previously treated CLL/SLL and MCL including covalent BTKi-resistance patients (129). Pirtobrutinib was used in 52 BTKi pre-treated MCL patients (ORR 52%) and it was well tolerated (130).

##### IMiDs: lenalidomide and pomalidomide

Lenalidomide and pomalidomide (POM) are next-generation IMiDs, and both have been shown the CNS permeability, especially pomalidomide was shown to have higher CNS penetration than lenalidomide (40% vs.11%) (56). The molecular mechanisms of IMiDs include inhibition of NF-κB and PI3K/AKT axis, as well as interferon regulatory factor 4 (IRF4) (131). Lenalidomide is active as monotherapy in relapsed PCNSL, and maintenance treatment prolongs response duration after salvage (54). The combination of POM and dexamethasone has significant therapeutic activity against R/R PCNSL and PVRL in 29 patients (ORR: 48%; median PFS: 5.3 months) (56).

##### XPO1 inhibitors: Selinexor

Selinexor (KPT-330) is a selective nuclear export inhibitor of exportin-1 (CRM1/XPO1). FOXO3a/PTEN-dependent resistance could be restored in ibrutinib-resistant cells by a nuclear export inhibitor (113). Selinexor was firstly reported as reversing resistance through the combination of ibrutinib and selinexor in CLL ibrutinib- refractory cells with BTK C481S mutation (132). The synergy effect was also observed for ibrutinib-resistant ABC-DLBCL cells (113). Selinexor crosses the BBB and favorable CNS penetration property was investigated with brain: plasma ratio 0.72 in rats (133). In pre-clinical mouse models of PCNSL, the combination of selinexor and ibrutinib was showed as promising therapeutic option (134).

##### Immune checkpoint inhibitors

Immune checkpoint molecules including PD-1 and PD-L1 may result in tumor cell escape in some solid tumors and hematological malignancy. Increased PD-L1 expression in tumor tissue or high PD-1 expression on tumor-infiltrating lymphocytes may predict treatment response to PD-1 blockade. High PD-L1 expression (>5% staining) was found in 18/48 patients (37.5%), PD-1 expression was found in 12/14 (85.7%) of PCNSL tumor specimens (135). In a study of 71 patients with PCNSL, PD-1 expression and PD-L1 expression were found in 16/71 and 42/71 patients, respectively (136). In the meanwhile, preclinical results indicate 50% of CNSL model mice treated with anti-PD1 achieved CR and potential cure in long-time surviving mice (137). Based on growing evidence, immune checkpoint inhibitors may be used as monotherapy or in combination with other treatments in R/R patients with PCNSL. In a case series study of five patients with R/R CNSL (4/5 PCNSL and 1/5 SCNSL), all patients achieved clinical response to PD-1 inhibitor nivolumab (CR:4 patients; PFS:13-17 months) (138). Almost the consistent treatment response was seen in Graber et al. study. All five patients (4/5 PCNSL and 1/5 SCNSL) were treated with PD-1 inhibitor pembrolizumab, two patients were observed durable remission (58). In Ambady et al. study, six R/R CNSL patients (3/6 PCNSL and 3/6 SCNSL) were treated with pembrolizumab in combination with rituximab, 3 patients achieved CR and maintained for more than six months (139). These small series studies support that immune checkpoint inhibitor could be a promising treatment option in CNS lymphoma.

#### BTK inhibitors adverse events

The adverse events of BTKis are often associated with immunosuppression and hematological toxicities including neutropenia, lymphopenia, anemia and thrombocytopenia, opportunistic infections such as aspergillosis and pneumocystis carinii pneumonia (PCP). In addition, BTKis also bind to other homologous kinases including TEC kinase family, interleukin-2-inducible T-cell kinase (ITK) and epidermal growth factor receptor (EGFR). The off-target effects of ibrutinib may induce atrial fibrillation, bleeding events, dermatological toxicities, diarrhoea, etc. According to the current literature, the adverse events of BTKi in PCNSL were summarized in **Table 2**.

**Table:** Table 2 BTK inhibitor adverse events.

BTKi	Literature	Enrolled Patients	Regimen	Adverse Events
ibrutinib	(18)	42 R/R PCNSL	ibrutinib 560-840mg	2 atrial fibrillations, 2 ventricular haemorrhages, 2 pulmonary aspergillosis, 2 anterior chamber haemorrhages
	(70)	13 R/R PCNSL	ibrutinib 560-840mg	20% grade 3-4 lymphopenia, 15% grade 3-4 neutropenia, 15% grade 3-4 hyperglycemia, 10% 3-4 grade thrombocytopenia
	(76)	5 ND PCNSL 13 R/R PCNSL	ibrutinib 560-840mg DA-TEDDi-R (16/18)	94% grade 4 neutropenia, 56% grade 4 thrombocytopenia, 50% pulmonary infections, 28% aspergillosis
	(77)	29 R/R PCNSL	ibrutinib 560-840mg	14% grade 3-4 lymphopenia, 11% grade 3-4 neutropenia, 9% grade 3-4 ALT elevation, 7% grade 3-4 hyperglycemia, 5% grade 3-4 thrombocytopenia
	(78)	9 R/R PCNSL	ibrutinib 560-840mg, HD-MTX and rituximab	20% lung infection, 7% grade 3-4 thrombocytopenia, 7% grade 3-4 hyperglycemia, 1% grade 3-4 neutropenia
	(80)	4 ND PCNSL 11 R/R PCNSL	ibrutinib 560-840mg, lenalidomide and rituximab	20% grade 3-4 lymphopenia, 20% grade 3 rash
	(81)	13 R/R PCNSL	ibrutinib 280-560mg DA-TEDDi-R (16/18)	82% grade 3-4 neutropenia, 30% grade 3-4 thrombocytopenia
zanubrutinib	(140)	13 R/R PCNSL	zanubrutinib 320mg**+** R/DHAP/MA/MTX ± R/DICE/CHOP/	38% grade 3-4 neutropenia, 8% grade 3-4 thrombocytopenia
orelabrutinib	(97)	13 R/R PCNSL	orelabrutinib 150mg**+** sintilimab	8% interstitial pneumonitis
tirabrutinib	(99)	44 R/R PCNSL	tirabrutinib 320-480mg	9.1% grade 3-4 neutropenia, 6.8% grade 3-4 lymphopenia, 4.5% grade 3-4 hyperglycemia, 4.5% grade 3-4 ALT elevation

## Conclusion

BTK inhibitor monotherapy or in combination with other treatments have shown good clinical efficacy in newly diagnosed and R/R PCNSL and are well tolerated, but BTKi resistance remains an unavoidable problem. The mechanisms of drug resistance and how to overcome drug resistance have been preliminarily explored, and some definite conclusions have been obtained. The combination of BTK inhibitors with other treatments may reverse BTKi resistance and have a long-term survival benefit in patients with PCNSL.

## Author contributions

All authors listed have made substantial and intellectual contribution in the preparation of the manuscript and final version was approved for publication.

## Conflict of interest

The authors declare that the research was conducted in the absence of any commercial or financial relationships that could be construed as a potential conflict of interest.

## Publisher’s note

All claims expressed in this article are solely those of the authors and do not necessarily represent those of their affiliated organizations, or those of the publisher, the editors and the reviewers. Any product that may be evaluated in this article, or claim that may be made by its manufacturer, is not guaranteed or endorsed by the publisher.
